# Hair cell maturation is differentially regulated along the tonotopic axis of the mammalian cochlea

**DOI:** 10.1113/JP279012

**Published:** 2019-12-21

**Authors:** Jing‐Yi Jeng, Federico Ceriani, Aenea Hendry, Stuart L. Johnson, Piece Yen, Dwayne D. Simmons, Corné J. Kros, Walter Marcotti

**Affiliations:** ^1^ Department of Biomedical Science University of Sheffield Sheffield S10 2TN UK; ^2^ Department of Biology Baylor University Waco TX 76798 USA; ^3^ School of Life Sciences University of Sussex, Falmer Brighton BN1 9QG UK

**Keywords:** action potentials, auditory, calcium signals, cochlea, development, hair cells

## Abstract

**Key points:**

- Outer hair cells (OHCs) enhance the sensitivity and the frequency tuning of the mammalian cochlea.
- Similar to the primary sensory receptor, the inner hair cells (IHCs), the mature functional characteristics of OHCs are acquired before hearing onset.
- We found that OHCs, like IHCs, fire spontaneous Ca^2+^‐induced action potentials (APs) during immature stages of development, which are driven by Ca_V_1.3 Ca^2+^ channels.
- We also showed that the development of low‐ and high‐frequency hair cells is differentially regulated during pre‐hearing stages, with the former cells being more strongly dependent on experience‐independent Ca^2+^ action potential activity.

**Abstract:**

Sound amplification within the mammalian cochlea depends upon specialized hair cells, the outer hair cells (OHCs), which possess both sensory and motile capabilities. In various altricial rodents, OHCs become functionally competent from around postnatal day 7 (P7), before the primary sensory inner hair cells (IHCs), which become competent at about the onset of hearing (P12). The mechanisms responsible for the maturation of OHCs and their synaptic specialization remain poorly understood. We report that spontaneous Ca^2+^ activity in the immature cochlea, which is generated by Ca_V_1.3 Ca^2+^ channels, differentially regulates the maturation of hair cells along the cochlea. Under near‐physiological recording conditions we found that, similar to IHCs, immature OHCs elicited spontaneous Ca^2+^ action potentials (APs), but only during the first few postnatal days. Genetic ablation of these APs *in vivo*, using *Ca_V_1.3^−/−^* mice, prevented the normal developmental acquisition of mature‐like basolateral membrane currents in low‐frequency (apical) hair cells, such as *I*_K,n_ (carried by KCNQ4 channels), *I*_SK2_ and *I*_ACh_ (α9α10nAChRs) in OHCs and *I*_K,n_ and *I*_K,f_ (BK channels) in IHCs. Electromotility and prestin expression in OHCs were normal in *Ca_V_1.3^−/−^* mice. The maturation of high‐frequency (basal) hair cells was also affected in *Ca_V_1.3^−/−^* mice, but to a much lesser extent than apical cells. However, a characteristic feature in *Ca_V_1.3^−/−^* mice was the reduced hair cell size irrespective of their cochlear location. We conclude that the development of low‐ and high‐frequency hair cells is differentially regulated during development, with apical cells being more strongly dependent on experience‐independent Ca^2+^ APs.

## Key points

- Outer hair cells (OHCs) enhance the sensitivity and the frequency tuning of the mammalian cochlea.
- Similar to the primary sensory receptor, the inner hair cells (IHCs), the mature functional characteristics of OHCs are acquired before hearing onset.
- We found that OHCs, like IHCs, fire spontaneous Ca^2+^‐induced action potentials (APs) during immature stages of development, which are driven by Ca_V_1.3 Ca^2+^ channels.
- We also showed that the development of low‐ and high‐frequency hair cells is differentially regulated during pre‐hearing stages, with the former cells being more strongly dependent on experience‐independent Ca^2+^ action potential activity.

## Introduction

The correct perception of acoustic stimuli in the mammalian cochlea depends on the sensory receptors, the inner and outer hair cells (IHCs and OHCs), and their respective afferent and efferent innervation (Goutman *et al*. 2015). While IHCs are the primary sensory receptors that relay sound information to type I afferent fibres, OHCs possess a unique combination of sensory and motor properties, which enhance the sensitivity and the frequency tuning within the cochlear partition (Dallos, 1992). Mature mouse OHCs express basolateral K^+^ channels with an unusually hyperpolarized activation (KCNQ4) (Kubisch *et al*. 1999; Marcotti & Kros, 1999). The current carried by these channels, *I*_K,n_, together with the resting depolarizing current flowing through the mechano‐electrical transducer channels, sets their resting membrane potential to near −40 mV (Johnson *et al*. 2011a). This relatively depolarized membrane potential ensures optimal activation of the motor protein prestin (Zheng *et al*. 2000; Liberman *et al*. 2002), which drives the somatic motility feedback mechanism characteristic of mature OHCs (Brownell *et al*. 1985; Ashmore, 1987). Another key feature of mature OHCs is that they are the primary target of the medial olivocochlear (MOC) efferent fibres (Liberman, 1980; Guinan *et al*. 1983; Simmons *et al*. 1996; Maison *et al*. 2003). Efferent fibres release the neurotransmitter acetylcholine (ACh), the role of which is to inhibit OHC electromotility and hence reduce the mechanical amplification of the cochlear partition (Guinan, 1996). OHC inhibition is achieved because ACh, by promoting Ca^2+^ influx through α9α10 nicotinic acetylcholine receptors (α9α10nAChRs), leads to the opening of the hyperpolarizing small conductance Ca^2+^‐activated K^+^ channels (SK2: Oliver *et al*. 2000; Katz *et al*. 2004; Lioudyno *et al*. 2004; Marcotti *et al*. 2004a).

OHCs from altricial rodents are very immature at birth and only begin to acquire the above biophysical and morphological characteristics of mature cells towards the end of the first postnatal week (He *et al*. 1994; Simmons *et al*. 1996; Oliver *et al*. 1997; Marcotti & Kros, 1999; Abe *et al*. 2007). On the other hand, IHCs start to acquire their mature basolateral current profile a few days later than OHCs, which corresponds to the onset of hearing at P12 (Kros *et al*. 1998; Marcotti *et al*. 2003a). Currently, it is not known whether the functional maturation of OHCs is solely dependent on an intrinsic genetic programme or can also be influenced by experience‐independent electrical activity similar to other sensory systems (e.g. Katz & Shatz, 1996; Blankenship & Feller 2010). Repetitive action potential activity has been shown to occur in the IHCs of the immature mouse cochlea (e.g. Kros *et al*. 1998; Tritsch *et al*. 2007; Johnson *et al*. 2011b, 2017; Sendin *et al*. 2014; Corns *et al*. 2018; Eckrich *et al*. 2018), which is used to influence the refinement of the exocytotic machinery (Johnson *et al*. 2013) and basolateral membrane currents (Brandt *et al*. 2003) in IHCs. OHCs appear less capable of firing spontaneous and repetitive AP activity in both mice and rats (Oliver *et al*. 1997; Marcotti & Kros, 1999; Weisz *et al*. 2012), although a previous study showed AP activity, but by using elevated extracellular Ca^2+^ and high intracellular EGTA (Beurg *et al*. 2008), and another has reported spontaneous Ca^2+^ signals during early postnatal stages (Ceriani *et al*. 2019).

Here we found that OHCs, under physiological recording conditions, are capable of firing spontaneous APs during early stages of development, just before the onset of functional maturation at ∼P7. Similar AP activity was previously described in IHCs, although this activity persists until near the onset of hearing at P12 (Johnson *et al*. 2011b, 2012). These APs are driven by Ca_V_1.3 Ca^2+^ channels. Using Ca_V_1.3 Ca^2+^ channel knockout mice (*Ca_V_1.3^−/−^*), we found that pre‐hearing Ca^2+^‐dependent APs are required for the normal acquisition of morphological and biophysical features that are characteristic of fully functional mature OHCs and IHCs. However, low‐frequency (apical coil) hair cells were more susceptible to the absence of pre‐hearing Ca^2+^ APs than high‐frequency (basal coil) cells, indicating a differential regulation of hair cell development along the cochlea. The expression of the motor protein prestin and the associated OHC electromotility were normal in mature *Ca_V_1.3^−/−^* mice, showing that experience‐independent Ca^2+^ signalling during immature stages does not influence the acquisition of key features of mature hair cells.

## Methods

### Ethics statement

All animal work was performed at the University of Sheffield and University of Sussex (UK) and licensed by the Home Office under the Animals (Scientific Procedures) Act 1986 and was approved by the University of Sheffield Ethical Review Committee. For *in vitro* work, mice were culled by cervical dislocation, which is a Schedule 1 method. *In vivo* recordings (ABR and DPOAE measurements: see below) were conducted under anesthesia using ketamine (100 mg kg^−1^, Fort Dodge Animal Health, Fort Dodge, IA, USA) and xylazine (10 mg kg^−1^, Rompun 2%, Bayer, HealthCare LLC, NY, USA), which were administered by intraperitoneal injection as previously described (Ingham *et al*. 2011). At the end of the experiments, which lasted up to 40 min, mice were culled using Schedule 1 methods.

### Tissue preparation

The study was performed on wild‐type and transgenic mice (*Ca_V_1.3^−/−^* mice: Platzer *et al*. 2000) of either sex on the C57BL/6 background. Apical‐ and basal‐coil hair cells were mainly studied in acutely dissected organs of Corti from postnatal day 0 (P0) to P18, where the day of birth (P0) corresponds to embryonic day 19.5 (E19.5). For some experiments in which recordings were performed from embryonic OHCs, mice were paired overnight and checked for vaginal plugs the following morning. Assuming ovulation occurs midway through the dark cycle, the mid‐point of the light cycle of the day following mating is considered to be E0.5 (Marcotti *et al*. 2003a). The genotyping protocol for the *Ca_V_1.3^−/−^* mice was performed as previously described (Platzer *et al*. 2000). Mice were killed by cervical dislocation and the organ of Corti dissected in extracellular solution composed of (in mm): 135 NaCl, 5.8 KCl, 1.3 CaCl_2_, 0.9 mgCl_2_, 0.7 NaH_2_PO_4_, 5.6 d‐glucose, 10 HEPES‐NaOH. Sodium pyruvate (2 mm), amino acids and vitamins were added from concentrates (Thermo Fisher Scientific, UK). The pH was adjusted to 7.5 (osmolality ∼308 mmol kg^−1^). For OHCs recorded *in situ*, the dissected organ of Corti was transferred to a microscope chamber, immobilized using a nylon mesh fixed to a stainless steel ring and viewed using an upright microscope (Olympus BX51, Japan; Leica, DMLFS, Germany; Nikon, Germany; Bergamo II System B232, Thorlabs Inc.). Hair cells were observed with Nomarski differential interference contrast (DIC) optics (×63 water immersion objective) or Dodt gradient contrast (DGC) optics (×60 water immersion objective) and either ×10 or ×15 eyepieces.

### Single‐cell electrophysiology

**Figure 1:**
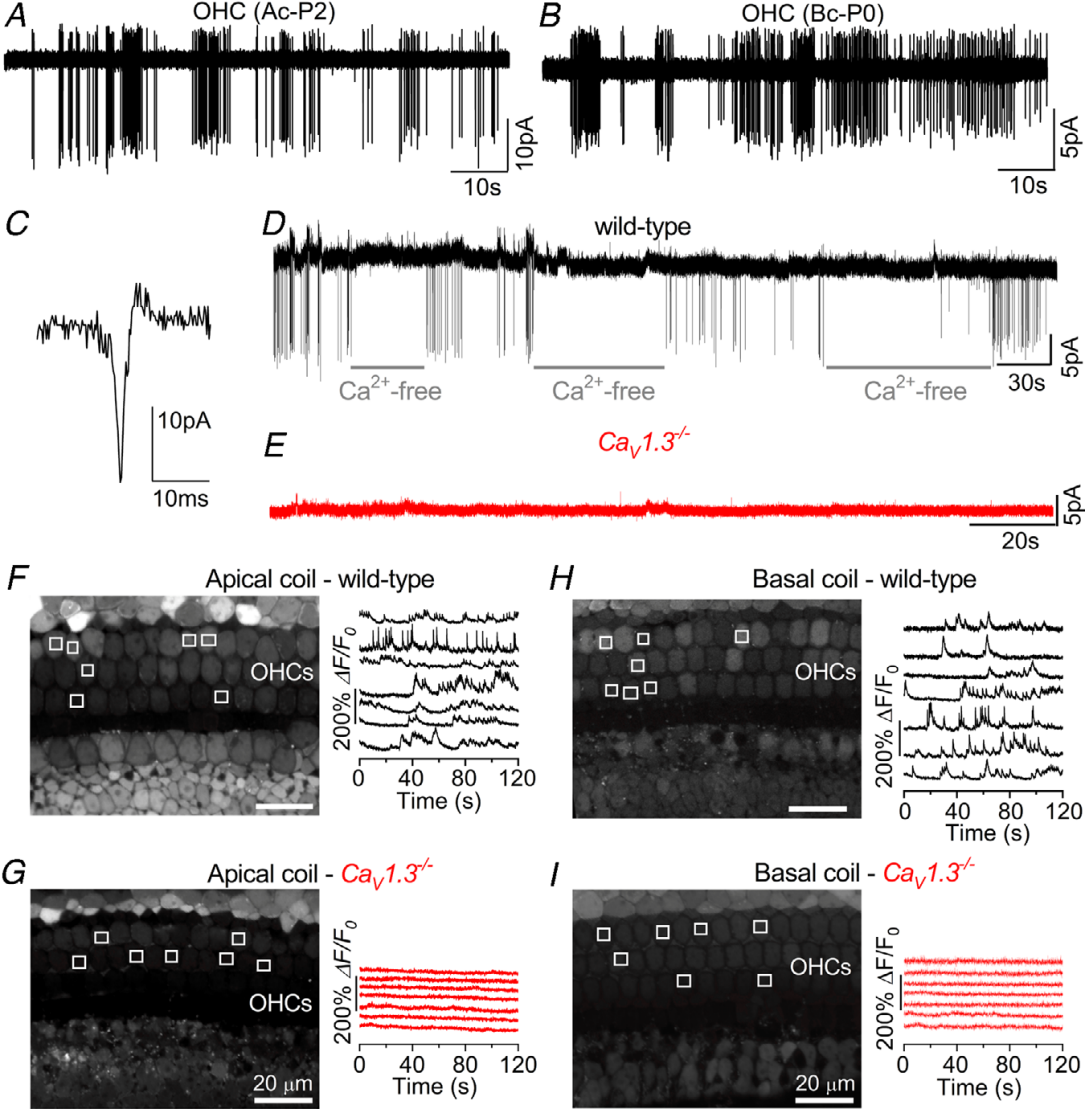
Early postnatal OHCs show spontaneous Ca^2+^‐induced action potentials *A* and *B*, spontaneous inward currents recorded from P2 apical coil (Ac) and P0 basal coil (Bc) OHCs, respectively, using cell‐attached voltage clamp at body temperature (∼35°C). Recordings were performed using physiological 1.3 mm extracellular Ca^2+^. *C*, expanded view of a single spontaneous current from panel *A*. *D*, cell‐attached recordings of inward currents from a P3 apical OHC during the repeated application of a Ca^2+^‐free solution containing 0.5 mm EGTA, a condition that abolishes APs (Johnson *et al*. 2011b). *E*, example of cell‐attached recording from a basal‐coil OHC of a P1 *Ca_V_1.3* ^−/−^ mouse with no spontaneous currents. *F*–*I*, representative Δ*F/F*_0_ traces from 7 apical (*F* and *G*) and basal (*H* and *I*) OHCs each of a P2 wild‐type (*F* and *H*) and a P1 *Ca_V_1.3^−/−^* (*G* and *I*) mouse. Traces are computed as pixel averages of regions of interest (white squares) centred on OHCs (Ceriani *et al*. 2019). Scale bars: 20 µm.

For cell‐attached recordings (Fig. 1), the pipette contained (in mm): 140 NaCl, 5.8 KCl, 1.3 CaCl_2_, 0.9 mgCl_2_, 0.7 NaH_2_PO_4_, 5.6 d‐glucose, 10 HEPES‐NaOH (pH 7.5; 308 mmol kg^−1^). For whole‐cell recordings, which were used in all the other figures containing electrophysiological data, the pipette intracellular solution contained (in mm): 131 KCl, 3 mgCl_2_, 1 EGTA‐KOH, 5 Na_2_ATP, 5 HEPES‐KOH, 10 sodium phosphocreatine (pH was adjusted with 1 m KCl to 7.28; 294 mmol kg^−1^). For Ca^2+^ current recordings, the pipette intracellular solution contained (in mm): 125 CsCl, 3 mgCl_2_, 1 EGTA‐CsOH, 5 Na_2_ATP, 5 HEPES‐CsOH, 5 TEA, 5 4‐AP (pH 7.3, 294 mmol kg^−1^). Membrane currents and voltage responses were investigated either at room temperature (20–24°C) or at body temperature (∼35°C), using Optopatch (Cairn Research Ltd, UK) or Axopatch 200B (Molecular Devices, USA) amplifiers. Data acquisition was controlled by pClamp software using Digidata 1320A, 1440A or 1550 boards (Molecular Devices). Recordings were low‐pass filtered at 2.5 kHz (8‐pole Bessel), sampled at 5 khz and stored on computer for off‐line analysis (Origin: OriginLab, USA). Membrane potentials in whole‐cell recordings were corrected for the residual series resistance *R*_s_ after compensation (usually 70–90%) and the liquid junction potential (LJP) of −4 mV measured between electrode and bath solutions. The extracellular application of a Ca^2+^‐free solution or solutions containing acetylcholine (Sigma‐Aldrich, UK) or strychnine (Tocris Bioscience, UK) was performed with a multi‐barrelled pipette positioned close to the patched cells.

The presence of electromotile activity in OHCs was estimated by applying a depolarizing voltage step from the holding potential of –84 mV to +36 mV and cell length was viewed with a Nikon FN1 microscope with ×75 magnification and images captured using a Flash 4.0 SCCD camera (Hamamatsu, Japan). Cell body movement was analysed using Fiji ImageJ software (Schindelin *et al*. 2012). Lines were drawn across the basal membrane of patched OHCs, perpendicular to the direction of cell motion, and a projected time‐based *z*‐stack of the pixels under the line was made. Cell movement was measured with Photoshop as a pixel shift and then converted to nanometres (290 pixels = 10 µm).

Non‐linear (voltage‐dependent) capacitance was measured from P11 hair cells using whole‐cell patch clamp recordings. In order to block most of the ion channels in hair cells, the pipette intracellular solution contained (in mm): 125 CsCl, 3 mgCl_2_, 1 EGTA‐CsOH, 5 Na_2_ATP, 5 HEPES‐CsOH, 5 tetraethylammonium (TEA), 5 4‐amynopyridine (4‐AP) (pH was adjusted with CsOH to 7.28; osmolality was 290 mmol kg^−1^). Real‐time changes in non‐linear membrane capacitance (*C*_N‐L_) were investigated using the capacitance track‐in‐mode of the Optopatch amplifier (Cairn Research Ltd, UK) during the application of a 4 kHz sine wave of 13 mV RMS. From the holding potential of −84 mV, hair cells were subjected to a voltage ramp from −154 mV to +96 mV over 2 s. The capacitance signal from the Optopatch amplifier was filtered at 250 Hz and sampled at 5 kHz.

### Two‐photon confocal Ca^2+^ imaging

For calcium dye loading, acutely dissected preparations were incubated for 40 min at 37°C in DMEM/F12, supplemented with fluo‐4 AM (final concentration 10–20 µM; Thermo Fisher Scientific). The incubation medium also contained pluronic F‐127 (0.1%, w/v, Sigma Aldrich, UK), and sulfinpyrazone (250 µM) to prevent dye sequestration and secretion. Preparations were then transferred to the microscope stage and perfused with extracellular solution for 20 min before imaging to allow for de‐esterification. Ca^2+^ signals were recorded using a two‐photon laser‐scanning microscope (Bergamo II System B232, Thorlabs Inc., USA) based on a mode‐locked laser system operating at 800 nm, 80‐MHz pulse repetition rate, < 100‐fs pulse width (Mai Tai HP DeepSee, Spectra‐Physics, USA). Images were formed by a ×60 objective, 1.1 NA (LUMFLN60XW, Olympus, Japan) using a GaAsp photomultiplier tube (Hamamatsu) coupled with a 525/40 bandpass filter (FF02‐525/40‐25, Semrock). Images were analysed off‐line using custom‐built software routines written in Python (Python 2.7, Python Software Foundation) and Fiji ImageJ software (Schneider et al. 2012). Ca^2+^ signals were measured as relative changes of fluorescence emission intensity (∆*F*/*F*_0_). Δ*F* = *F* – *F*_0_, where *F* is fluorescence at time *t* and *F*_0_ is the fluorescence at the onset of the recording

### Immunofluorescence microscopy

Dissected inner ears from wild‐type and *Ca_V_1.3^−/−^* mice (*n* = 4 for each set of experiment) were fixed with 4% paraformaldehyde in phosphate‐buffered saline (PBS, pH 7.4) for 5–20 min at room temperature. Cochleae were microdissected, rinsed three times for 10 min in PBS, and incubated for 1 h at room temperature in PBS supplemented with 5% normal goat or horse serum and 0.5% Triton X‐100. The samples were then incubated overnight at 37°C with the primary antibody in PBS supplemented with 1% of the specific serum. Primary antibodies were: mouse anti‐myosin 7a (1:1000, Developmental Studies Hybridoma Bank, No. 138‐1C), rabbit anti‐myosin 7a (1:200, Proteus Biosciences, No. 25‐6790), rabbit anti‐prestin (1:5000, kindly provided by Robert Fettiplace), mouse anti‐KCNQ4 (1:100, StressMarq, No. SMC‐309D), goat anti‐Oncomodulin (1:200, Swant, OMG4), rabbit anti‐SK2 (1:500, Sigma‐Aldrich, P0483), mouse anti‐BK (1:100, Antibodies, 75–408) and goat anti‐choline acetyltransferase (ChAT, 1:500, Millipore, AB144P). All primary antibodies were labelled with species appropriate Alexa Fluor secondary antibody for 1 h at 37°C. Samples were then mounted in VECTASHIELD. The *z*‐stack images were captured with a Nikon A1 confocal microscope equipped with Nikon CFI Plan Apo ×60 oil objective, which is part of the Light Microscope Facility at the University of Sheffield. Image stacks were processed with Fiji ImageJ Analysis software. Cell volume was estimated from the *z*‐stack images of the immunolabelled hair cells with the cell marker Myo7a using Imaris software (Oxford Instruments).

#### Auditory brainstem responses

Auditory brainstem responses (ABRs) were recorded from male and female wild‐type and *Ca_V_1.3^−/−^* mice between P16 and P18. Recordings were performed in a soundproof chamber (MAC‐3 Acoustic Chamber, IAC Acoustic, UK) as previously described (Ingham *et al*. 2011). Briefly, stimuli were delivered to the ear by calibrated loudspeakers (MF1‐S, Multi Field Speaker, Tucker‐Davis Technologies, USA) placed 10 cm from the animal's pinna. Sound pressure was calibrated with a low‐noise microphone probe system (ER10B+, Etymotic, USA). Experiments were performed using customized software (Ingham *et al*. 2011) driving an RZ6 auditory processor (Tucker‐Davis Technologies). Response thresholds were estimated from the resulting ABR waveform and defined as the lowest sound level where any recognizable feature of the waveform was visible. Responses were measured for click and stimulus pure tones of frequencies between 3 and 42 kHz. Stimulus sound pressure levels were typically 0–95 dB SPL, presented in steps of 5 dB. The brainstem response signal was averaged over 256 repetitions. Tone bursts were 5 ms in duration with a 1 ms on/off ramp time, which was presented at a rate of 42.6 s^−1^.

### Distortion product otoacoustic emissions

OHC function was assessed *in vivo* by measuring distortion product otoacoustic emissions (DPOAEs). Recordings were performed in a soundproof chamber (MAC‐3 Acoustic Chamber, IAC Acoustic, UK). DPOAEs were recorded at 2f1‐f2 in response to primary tones of frequency f1 and f2, where f2/f1 = 1.2. The f2 level (L2) was set from 20 to 80 dB with 10 dB increments, and the f1 level (L1) was set equal to L2. Frequency pairs of tones between f2 = 6 kHz and f2 = 36 kHz were presented directly into the ear canal by means of a metal coupler connected to two calibrated loudspeakers (MF1‐S, Multi Field Speaker, Tucker‐Davis Technologies, USA). The emission signals were recorded by a low‐noise microphone (ER10B+: Etymotic Research Inc, USA) connected to the coupler. Experiments were performed using BioSigRZ software driving an RZ6 auditory processor (Tucker‐Davis Technologies). The DPOAE thresholds were defined by the minimal sound level, where the DPOAEs were two times above the standard deviation of the noise. The determined DPOAE thresholds were plotted against the geometric mean frequency of f1 and f2. Stimulus sound pressure levels were typically 20–80 dB SPL, presented in steps of 10 dB. The response signal was averaged over 500 repetitions.

### Statistical analysis

Statistical comparisons of means were made by Student's two‐tailed *t* test or, for multiple comparisons, analysis of variance (one‐way or two‐way ANOVA followed by Bonferroni's or Tukey's *post hoc* test) was applied. *P < *0.05 was selected as the criterion for statistical significance. Mean values are quoted as means ± SD (ABR and DPOAE experiments) or means ± SEM (all other figures).

## Results

### Immature OHCs fire spontaneous Ca^2+^‐dependent action potentials

Using cell‐attached patch clamp, which preserves the *in vivo* intracellular milieu and Ca^2+^ levels, we recorded spontaneous action potentials (APs) in the form of biphasic capacitative currents (Fig. 1 *A*–*C*; see also Johnson *et al*. 2011b) in early postnatal apical (Fig. 1 *A*) and basal (Fig. 1 *B*) OHCs. We compared the AP activity of P2 apical and P0 basal OHCs because the development of the former lags behind that of basal cells by about 2 days (Helyer *et al*. 2005: see also Fig. 4). The mean spike frequency of apical OHCs (0.75 ± 0.13 Hz, *n* = 7) was significantly lower than that in basal cells (1.95 ± 0.34 Hz, *n* = 4, *P = *0.0033, *t* test). However, the coefficient of variation (CV) was not significantly different (apical: 1.62 ± 0.18, *n* = 7; basal: 1.77 ± 0.28, *n* = 4, *P = *0.6506; *t* test), and, being greater than one, is indicative of a bursting pattern of activity. Action potentials were mainly recorded from early postnatal OHCs and by P6 they were no longer present, which agrees with previous measurements of Ca^2+^ signals using 2‐photon imaging (Ceriani *et al*. 2019). Spontaneous AP activity was abolished by the perfusion of a Ca^2+^‐free solution (Fig. 1 *D*) and was absent in *Ca_V_1.3^−/−^* mice (Fig. 1 *E*). Spontaneous, rapid Ca^2+^ signals were also recorded from control OHCs maintained in acutely dissected cochleae loaded with the Ca^2+^ indicator Fluo‐4 (Fig. 1 *F* and *H*). These Ca^2+^ signals represent the optical read‐out of OHC firing activity in wild‐type mice (see also Ceriani *et al*. 2019) and were absent in both apical and basal cells of *Ca_V_1.3^−/−^* mice (Fig. 1 *G* and *I*).

### OHCs from *Ca_V_1.3^−/−^* mice retain healthy characteristics up to the onset of hearing

**Figure 2:**
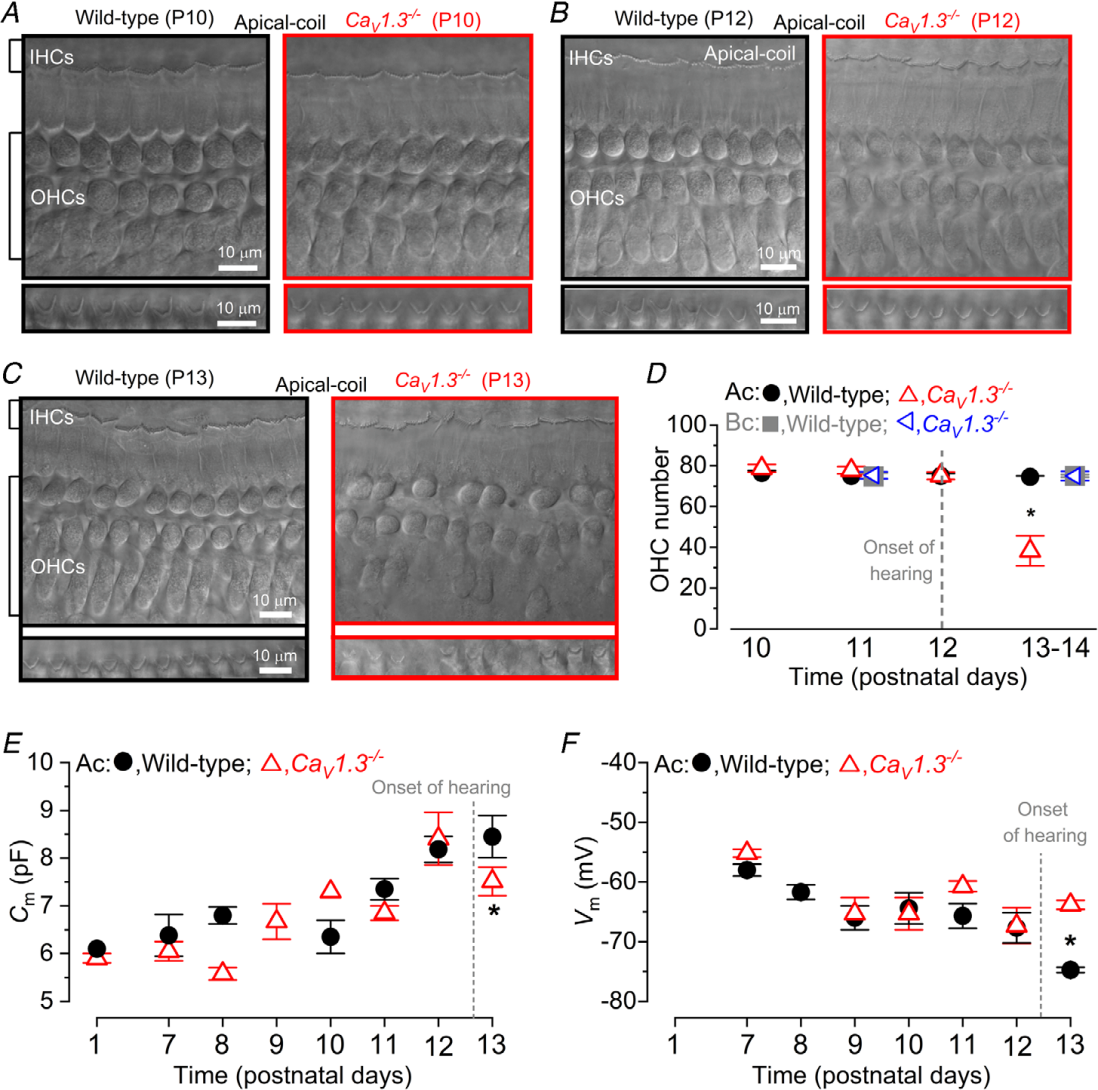
The number and size of pre‐hearing OHCs is normal in *Ca_V_1.3^−^^/^^−^* mice *A* and *B*, DIC images of a region of the mouse cochlear apical coil (∼8–12 kHz) showing the presence of IHCs and OHCs at P10 and P12 for wild‐type (left top panels) and *Ca_V_1.3^−/−^* (right top panels) mice. Note that the hair bundles of IHCs are also visible. The presence of normal hair bundles in OHCs is shown in the bottom panels. *C*, DIC images from a wild‐type (left) and *Ca_V_1.3^−/−^* (right) mice, as described in panels *A* and *B*, but from a cochlea just after hearing onset (P13). Note that OHCs began to disappear in *Ca_V_1.3^−/−^* mice. *D*, number of OHCs present in a 200 µm region from the apical and basal coil of wild‐type and *Ca_V_1.3^−/−^* mice as a function of postnatal development. Numbers of cochleae tested at the various ages from left to right are: wild‐type apical 6, 7, 6, 6; *Ca_V_1.3^−/−^* apical 4, 6, 7, 5. ^*^ *P < *0.0001; wild‐type basal 3, 3; *Ca_V_1.3^−/−^* basal 4, 4. *E*, membrane capacitance (*C*_m_) measured from apical OHCs of wild‐type and *Ca_V_1.3^−/−^* mice during development. Numbers of OHCs measured: wild‐type 3, 6, 5, 0, 2, 9, 23, 4; *Ca_V_1.3^−/−^* 4, 4, 5, 7, 1, 2, 10, 14. ^*^ *P*<0.0001. *F*, resting membrane potential (*V*_m_) of wild‐type and *Ca_V_1.3^−/−^* OHCs during development. ^*^ *P = *0.0013. Numbers of OHCs measured: wild‐type 3, 4, 2, 4, 8, 6, 4; *Ca_V_1.3^−/−^* 3, 2, 2, 4, 3, 7. P11 data points are from Ceriani *et al*. 2019.

Previous light microscopy studies have shown that apical‐coil OHCs from *Ca_V_1.3^−/−^* mice are present at P7 but largely absent at P14–P15, with their degeneration progressing from the apical to the basal region of the cochlea (Platzer *et al*. 2000; Glueckert *et al*. 2003). Therefore, we initially investigated the progression of OHC degeneration in the apical region used for the majority of our experiments, which corresponded to a frequency range in the adult mouse of ∼8–12 kHz (Müller *et al*. 2005; see also Ceriani *et al*. 2019). Cochleae from wild‐type and *Ca_V_1.3^−/−^* mice between P10 and P14 were acutely dissected and DIC images were taken from the apical coil. The appearance (e.g. hair bundle and basolateral region) and number of apical‐coil OHCs was similar between the two genotypes up to P12 (Fig. 2 *A*, *B* and *D*). However, from just after the onset of hearing, P13–P14 apical‐coil OHCs from *Ca_V_1.3^−/−^* mice exhibited an abrupt reduction in number compared to wild‐type mice (Fig. 2 *C* and *D*: *P < *0.0001, Bonferroni's *post hoc* test, two‐way ANOVA). By contrast, the number of OHCs positioned in the basal coil of the cochlea, which corresponds to an approximate frequency range of ∼ 25–45 kHz (Müller *et al*. 2005; Ceriani *et al*. 2019), were not significantly different between wild‐type and *Ca_V_1.3^−/−^* mice at P11 (*P = *0.874) and after the onset of hearing (P14, *P ≥ *0.999, Fig. 2 *D*).

**Figure 3:**
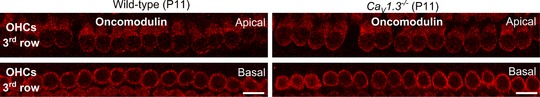
The Ca^2+^ binding protein oncomodulin is present in OHCs from wild‐type and *Ca_V_1.3^−^^/^^−^* mice Confocal images taken from apical and basal coil OHCs of wild‐type (left panel) and *Ca_V_1.3^−/−^* (right panel) mice at P11, which is after their onset of functional maturation at P8, but just before the onset of hearing (P12). Similar staining was seen in additional 4 mice for each genotype. Scale bar: 10 µm.

We then investigated two crucial biophysical properties of OHCs, their membrane capacitance (*C*_m_), which provides an indication of the cell surface, and resting membrane potential (*V*_m_), by performing whole‐cell patch clamp recordings. These experiments were only performed in apical OHCs because of the difficulty of obtaining reliable recordings from basal OHCs after P8. The OHC membrane capacitance, which provides a quantifiable measure of the cell's surface area (*C*_m_: Fig. 2 *E*) was not significantly different between the two genotypes (P1–P13: *P = *0.754, two‐way ANOVA), although a significant decrease in *C*_m_ was present in OHCs from *Ca_V_1.3^−/−^* mice at P13 (*P < *0.0001, Bonferroni's *post hoc* test). We then performed current clamp experiments in order to investigate the resting membrane potential (*V*_m_) of OHCs as a function of age, which provides a reliable measure of the baseline physiological status of cells. We found that the resting *V*_m_ was comparable between wild‐type and *Ca_V_1.3^−/−^* OHCs up to P12, becoming significantly more depolarized only at P13 in the latter (Fig. 2 *F*, *P = *0.0013, Bonferroni's *post hoc* test, two‐way ANOVA). Another key functional marker of OHCs is the presence of oncomodulin, which is a member of the parvalbumin family of Ca^2+^ binding proteins expressed in OHCs from early postnatal ages (Simmons *et al*. 2010). We found that oncomodulin was present in both apical and basal OHCs from P11 wild‐type and *Ca_V_1.3^−/−^* mice (Fig. 3).

The above morphological and biophysical data, including the electromotile activity (see below), demonstrate that OHCs from *Ca_V_1.3^−/−^* mice maintain a healthy functional profile for at least 5 days after their initial onset of maturation at the beginning of the second postnatal week. However, just after the onset of hearing at ∼P12, apical but not basal OHCs abruptly begin to degenerate.

### Apical OHCs from *Ca_V_1.3^−/−^* mice fail to acquire the full adult biophysical profile

**Figure 4:**
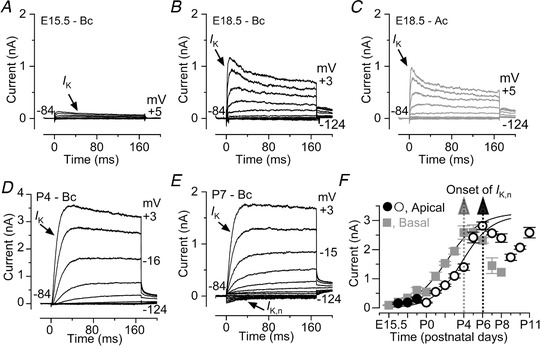
Acquisition of *I*_K,n_ in developing OHCs *A*–*E*, typical current responses from E15.5, E18.5, P4 and P7 OHCs recorded from wild‐type mice. Note that most of the examples are from basal‐coil OHCs apart from the recordings in panel *C* (see panel *F* below for details). Also note that the *y*‐axis in panel *D* covers a larger current range than that in all the other panels (*A*–*C* and *E*). Outward current was elicited by using depolarizing and hyperpolarizing voltage steps (10 mV increments) from –84 mV to the various test potentials shown by some of the traces. *F*, size of the total outward K^+^ current measured at 0 mV as a function of embryonic and early postnatal ages for OHCs positioned in the apical and basal cochlear region (including those shown in panels *A–E*). Note that the onset of *I*_K,n_ occurs about 2 days earlier in basal OHCs. Numbers of basal OHCs measured at the various ages (E15.5–P8) are from left to right: 3, 4, 5, 3, 3, 4, 27, 15, 13, 3, 7, 4, 1; apical OHCs (E16.5–E18.5) 5, 4, 4 and (P0–P11) are from Marcotti & Kros (1999). Fits to the data are according to a sigmoidal logistic growth curve: $A=A_{\min}+\frac{\left(A_{\max}-A_{\min}\right)}{1+\exp(-k(t-t_{\mathrm{half}}))},$ where *A* is the size of the current, *k* is a slope factor and *t*_half_ is the age where *A* is halfway between the maximal (*A*_max_) and minimal (*A*_min_) currents. *t*_half_ was 2.2 days in basal and 3.8 days in apical OHCs.

Since the presence of early spontaneous AP activity has been shown to regulate the transcription of genes involved in cell development (e.g. Greer & Greenberg, 2008), we investigated whether OHCs from *Ca_V_1.3^−/−^* mice, where Ca^2+^ spikes are absent, were able to acquire their normal adult‐like characteristics. OHC fate is determined during early embryonic development and from about embryonic day 14 (E14) they are recognizable within the sensory epithelium (Pujol *et al*. 1998). Using whole‐cell patch clamp recordings, we compared the developmental acquisition of the K^+^ currents in mouse OHCs (Fig. 4 *A–F*). Small outward K^+^ currents (*I*_K_) were recorded as early as E15.5 in basal and E16.5 in apical OHCs. Their amplitude gradually increased with age and reached a maximum at around P4 in basal and P6 in apical OHCs (Fig. 4 *F*), at about the time when OHCs lose their ability to fire spontaneous Ca^2+^ spikes. During the following few days the size of the immature *I*_K_ gradually decreased (Fig. 4 *F*). At the same time OHCs began to express a negatively activating K^+^ current *I*_K,n_ (Fig. 4 *E*), the main K^+^ current in mature mouse OHCs (Marcotti & Kros, 1999). This caused the overall outward K^+^ current to increase again (Fig. 4 *F*), reaching a mature‐like amplitude in apical OHCs at around P12 (Marcotti & Kros, 1999). A similar developmental change in the K^+^ currents is also likely to occur in basal OHCs but, as mentioned above, patch‐clamp recordings in basal cells became unreliable after P8.

**Figure 5:**
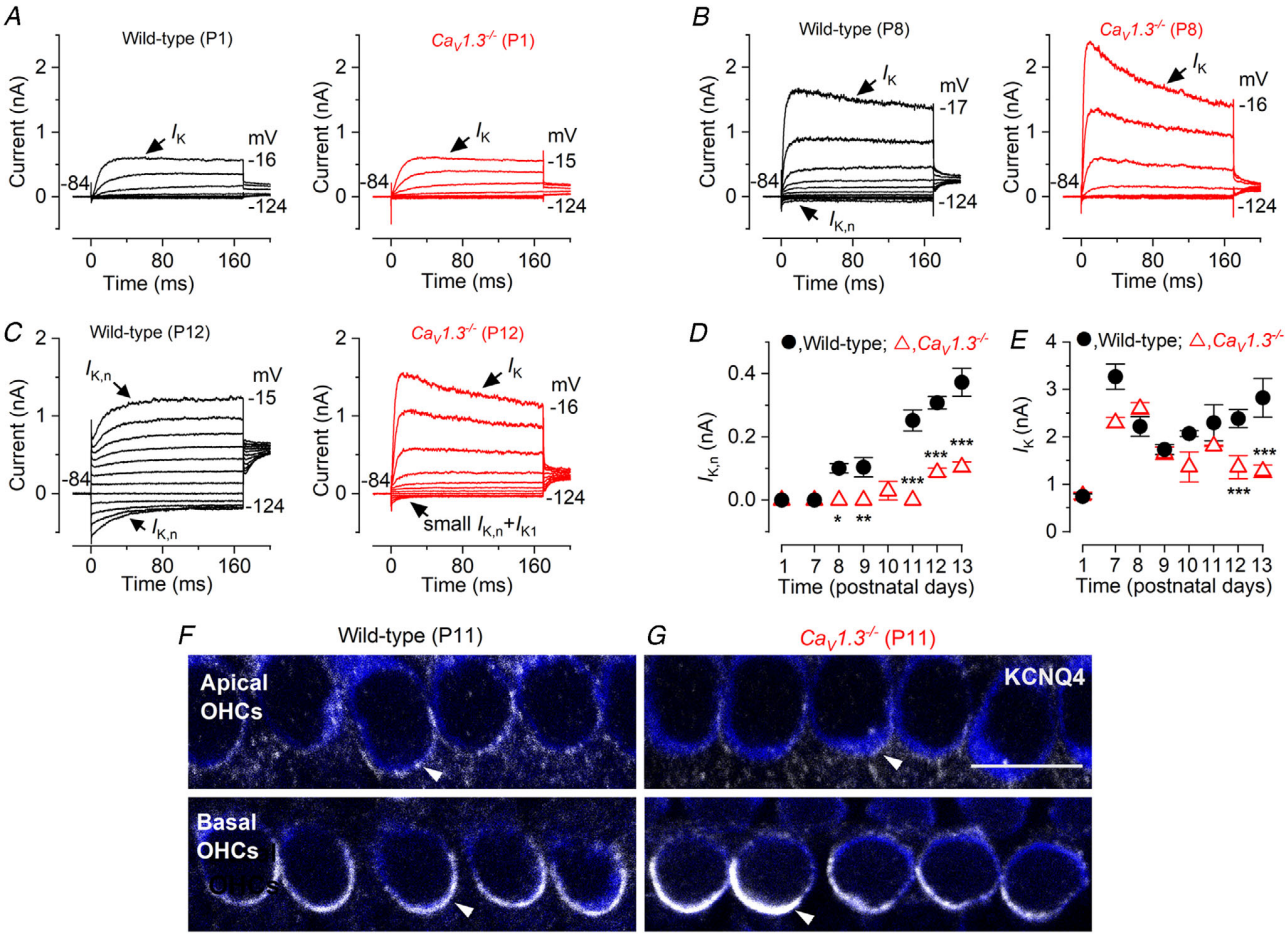
The expression of the mature K^+^ current profile is impaired in *Ca_V_1.3* *^−/−^* OHCs *A*–*C*, current responses in wild‐type (left) and *Ca_V_1.3^−/−^* (right) apical‐coil OHCs during immature stages (*A*, P1), at the onset of function when *I*_K,n_ is first detected (*B*, P8: Marcotti & Kros, 1999) and at a more mature age when *I*_K,n_ has almost reached its mature size in mice (*C*, P12: Marcotti & Kros, 1999). Note that even at P12 *I*_K,n_ is very small and possibly contaminated by the inward rectifier *I*_K1_. *D*, size of the isolated *I*_K,n_ as a function of postnatal age in wild‐type and *Ca_V_1.3^−/−^* apical OHCs (measured as the deactivating tail currents at −124 mV from the holding potential of −84 mV). Numbers of cells from left to right: wild‐type: 3, 7, 5, 7, 0, 3, 15, 4; *Ca_V_1.3^−/−^* 4, 4, 4, 7, 3, 2, 12, 11. Two‐way ANOVA with Bonferroni's *post hoc* test: ^*^ *P = *0.0192 (P8); ^**^ *P = *0.0022 (P9), ^***^ *P < *0.0001 (at P11, P12 and P13). *E*, total steady‐state outward *I*_K_ as a function of age in wild‐type and *Ca_V_1.3^−/−^* OHCs (measured at 0 mV from the holding potential of −84 mV). ^***^ *P = *0.0002 (at P12 and P13). Number of OHCs investigated as in panel *D*. *F* and *G*, maximum intensity projections of confocal *z*‐stack images that were taken from apical (upper panels) and basal (lower panels) coil OHCs (P11) in wild‐type (*F*) and *Ca_V_1.3^−/−^* (*G*) mice. Immunostaining for KCNQ4 is shown in white (arrows) and Myo7a is used as a cell marker (blue). Similar staining was seen in an additional 4 mice for each genotype. Scale bar: 10 µm.

During the first postnatal week, apical‐coil OHCs from both wild‐type and *Ca_V_1.3^−/−^* mice were indistinguishable in terms of their basolateral membrane properties under whole‐cell patch clamp (Fig. 5 *A*, *D* and *E*). During the second postnatal week, wild‐type OHCs began to express *I*_K,n_, which becomes the major adult‐type K^+^ current by P12 as mentioned above (Fig. 5 *C*; see also Marcotti & Kros, 1999). On the other hand, OHCs (≥P7) from *Ca_V_1.3^−/−^* mice retained the immature delayed rectifier as the main K^+^ current (Fig. 5 *C*), which was evident by the slower activating and inactivating time course (Marcotti & Kros, 1999). *I*_K,n_ in mature OHCs from *Ca_V_1.3^−/−^* mice was either absent or very small compared to that in wild‐type cells (Fig. 5 *D*, *P < *0.0001, two‐way ANOVA). The failure in the normal up‐regulation of *I*_K,n_ caused the total outward current to remain at a nearly constant size between P9 and P13 (Fig. 5 *E*, *P* = 0.628, one‐way ANOVA) but significantly lower than that in wild‐type OHCs (Fig. 5 *E*, *P* = 0.0003, two‐way ANOVA). Because OHCs from *Ca_V_1.3^−/−^* mice retain an immature‐like current profile, the measured *I*_K,n_ in these cells is likely to be contaminated by the inward rectifier K^+^ current *I*_K1_. This is because *I*_K1_ is normally expressed by immature OHCs and is strongly activated at the membrane potential used to estimate the size of *I*_K,n_ (Marcotti *et al*. 1999). The persistence of *I*_K1_ in *Ca_V_1.3^−/−^* OHCs would also explain why during pre‐hearing stages they exhibit a resting *V*_m_ comparable to that of wild‐type cells (Fig. 2 *F*), since *I*_K1_ is active at the resting *V*_m_ and fulfils a similar role to *I*_K,n_ in setting the resting *V*_m_ (Marcotti *et al*. 1999). Therefore we investigated the presence of KCNQ4 channels, which carry *I*_K,n_ in OHCs (Kubisch *et al*. 1999; Marcotti & Kros, 1999), using immunohistochemistry experiments. In P11 wild‐type mice, KCNQ4 staining was present throughout the entire basolateral membrane of both apical and basal OHCs, although the latter were more intensely stained (Fig. 5 *F*, e.g. white arrowheads). In age‐matched *Ca_V_1.3^−/−^* mice, KCNQ4 expression was much reduced in apical OHCs (Fig. 5 *G*, upper panel), supporting the electrophysiological data (Fig. 5 *D*), while it was easily detectable in basal OHCs (Fig. 5 *G*, lower panel).

### Prestin expression and electromotility are unaffected by the absence of spontaneous AP activity

**Figure 6:**
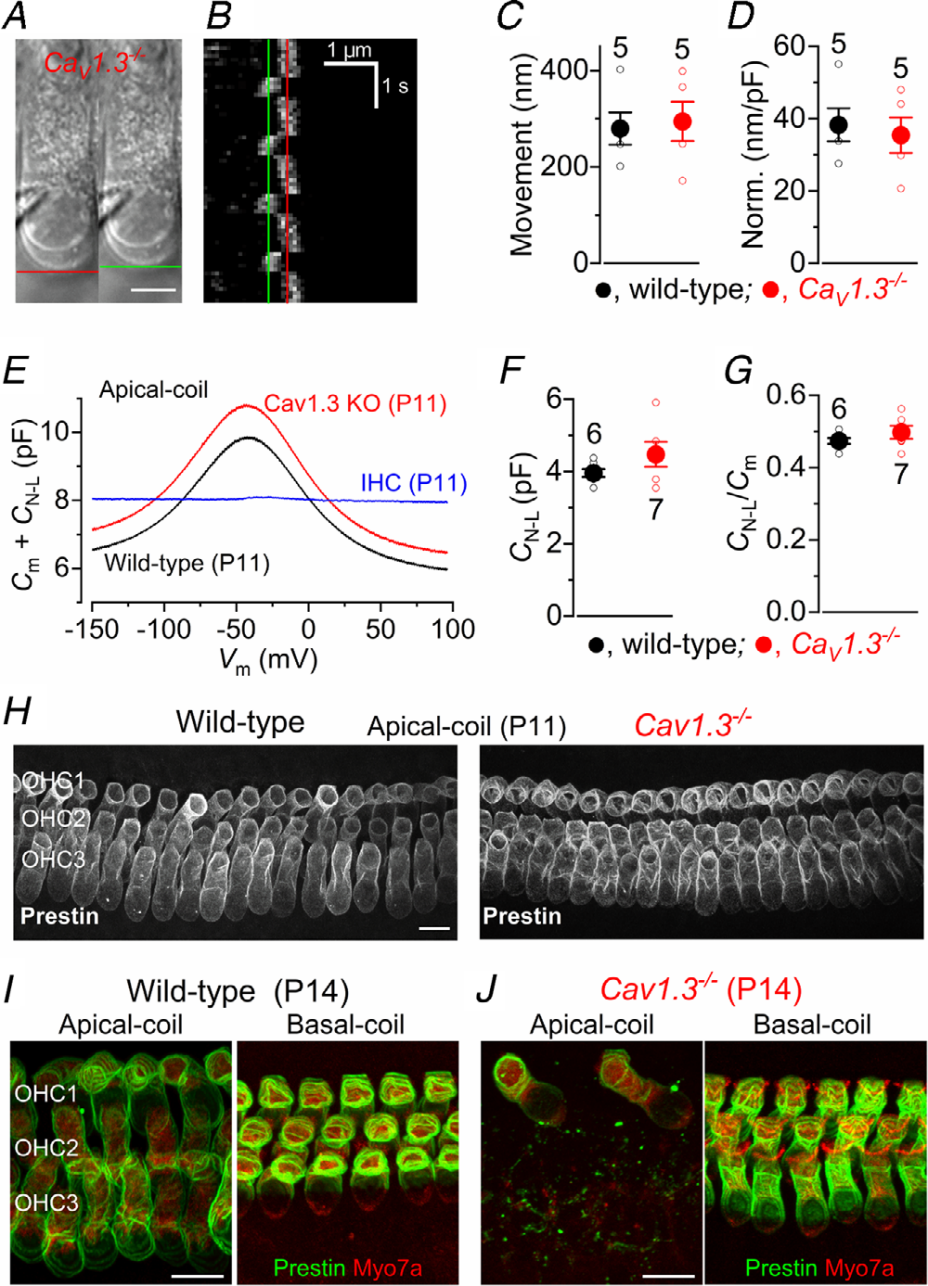
Prestin expression and electromotility are normal in OHCs from *Ca_V_1.3* *^−/−^* mice *A*, images showing the patch pipette attached to an apical‐coil OHC from a *Ca_V_1.3^−/−^* mouse at P12. The red lines indicate the position of the OHC basal membrane before (left: at –84 mV) and during a depolarizing voltage step from –84 mV to +36 mV (right). Note that membrane depolarization causes OHCs to shorten. Scale bar, 5 µm. *B*, kymograph showing the movement of the subnuclear region of the OHC shown in panel *A* as a function of time in response to the repetitive voltage steps described above. The red line indicates the resting position of the OHC basal membrane and the green line indicates the shortening. *C* and *D*, average movement in response to the depolarizing voltage step was not significantly different between wild‐type and *Ca_V_1.3^−/−^* apical‐coil OHCs (*C*, *P = *0.79), even after normalization to the average OHC membrane capacitance (*D*, *P = *0.68). *E*, examples of voltage‐dependent non‐linear capacitance (*C*_N‐L_) recorded in apical‐coil hair cells by applying a voltage ramp from –154 mV to +96 mV over 2 s. Note that the cell membrane capacitance (*C*_m_) was added to the measured *C*_N‐L_. *C*_N‐L_ was present in the OHCs from both genotypes, but absent in the IHC. *F* and *G*, average *C*_N‐L_ was not significantly different between wild‐type and *Ca_V_1.3^−/−^* apical‐coil OHCs (*F*), even after normalization to the individual OHC membrane capacitance *C*_m_ (*G*). *C*_N‐L_ was calculated as the difference between the peak of the recording near −40 mV and the lowest value at positive membrane potentials. Recordings in *A–G* are at room temperature. Number of cells investigated is shown near the columns. *H*, maximum intensity projections of confocal *z*‐stacks taken from the apical cochlear region of wild‐type (left) and *Ca_V_1.3^−/−^* (right) mice at P11 using antibodies against prestin (white). *I* and *J*, images obtained as in *H*, but from the apical (left panels) and basal (right panels) cochlear of wild‐type (*I*) and *Ca_V_1.3^−/−^* (*J*) post‐hearing mice (P14). The hair‐cell marker Myo7a (red) was used to better identify the synaptic, basal portion of OHCs that does not contain prestin (green); this was more evident in basal OHCs. Note that the different angle of the basal OHCs gives the incorrect impression that wild‐type OHCs are smaller than those in the *Ca_V_1.3^−/−^* mice. Prestin labelling was also present in the few remaining OHCs of *Ca_V_1.3^−/−^* mice (*J*, left panel). Scale bars in *H–J*: 10 µm.

The maturation of OHCs is also associated with the expression of the motor protein prestin (Zheng *et al*, 2000; Liberman *et al*, 2002), which drives the somatic motility of mouse OHCs from about P7 onwards (Marcotti & Kros, 1999; Abe *et al*. 2007). In line with the normal morphological appearance throughout pre‐hearing stages (≤P12: Fig. 2), OHCs from *Ca_V_1.3^−/−^* mice showed normal electromotile activity at P12 (Fig. 6). Under whole‐cell patch clamp conditions, stepping the membrane potential from −84 mV to +36 mV caused OHCs from both wild‐type and *Ca_V_1.3^−/−^* mice to shorten (Fig. 6 *A* and *B*), as previously described (Marcotti & Kros, 1999; Abe *et al*. 2007). OHC movement was not significantly different between the two genotypes (*P* = 0.7881: Fig. 6 *C* and *D*). This was further investigated using non‐linear (voltage‐dependent) capacitance changes (*C*_N‐L_), which is the electrical signature of electromotility in OHCs. We found that the maximum size of *C*_N‐L_ in P11 apical coil OHCs (Fig. 6 *E–G*) was comparable to that reported from P10–P12 cells (Abe *et al*. 2007), and was not significantly different between the two genotypes (*P = *0.1537: Fig. 6 *F*), even when it was normalized to the OHC membrane capacitance (*P = *0.2368: Fig. 6 *G*). The above electrophysiological results (Fig. 6 *A–D*: electromotility; Fig. 6 *E–G*: non‐linear capacitance) are in agreement with the normal expression of prestin in age‐matched OHCs (P11) from both wild‐type and *Ca_V_1.3^−/−^* mice (Fig. 6 *H*). The expression of prestin was also comparable between the two genotypes even after the onset of hearing (P14: *Fig*. 6 *I* and *J*), in the few remaining apical OHCs.

### Efferent synapses do not fully develop in apical OHCs from *Ca_V_1.3^−/−^* mice

OHC maturation is also associated with their ability to respond to the inhibitory efferent neurotransmitter acetylcholine (ACh) from around the end of the first postnatal week (Katz *et al*. 2004; Marcotti *et al*. 2004a), which coincides with the appearance of *I*_K,n_ (Marcotti & Kros, 1999). ACh exerts an overall inhibitory effect because Ca^2+^ that enters through α9α10nAChRs (Katz & Elgoyhen, 2014) then opens small‐conductance Ca^2+^ activated K^+^ channels (SK2 channels: Katz *et al*. 2004; Marcotti *et al*. 2004a), which leads to OHC hyperpolarization.

**Figure 7:**
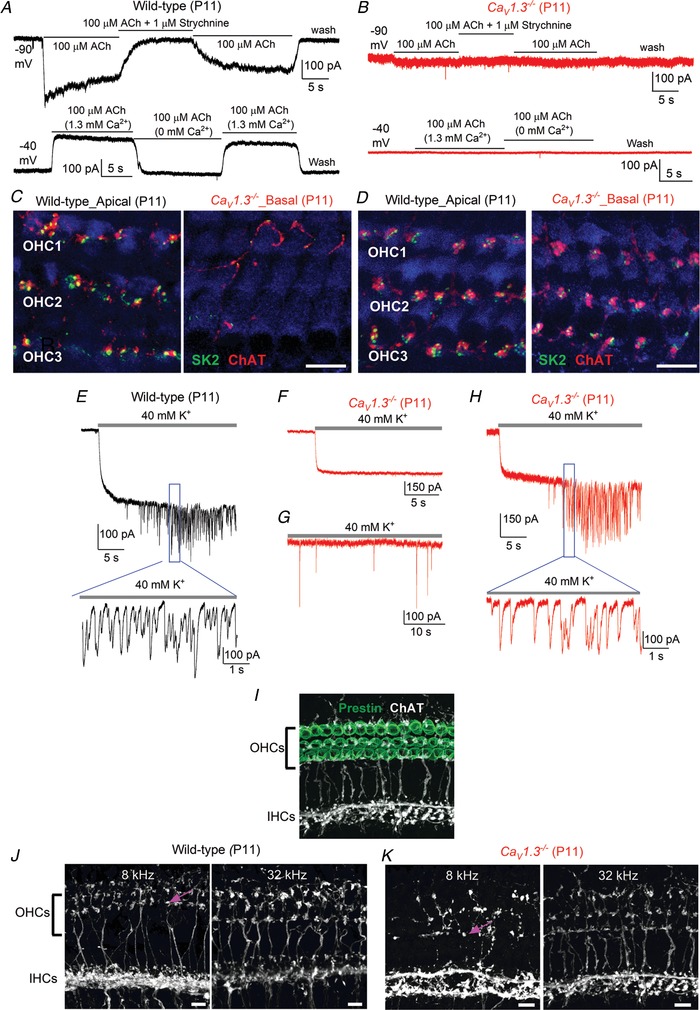
Cholinergic efferent synapses are differentially affected in apical and basal OHCs of *Ca_V_1.3^−^^/^^−^* mice *A*, inward (top) and outward (bottom) currents in wild‐type apical‐coil OHCs elicited during the extracellular application of 100 µM extracellular ACh at –90 mV and –40 mV, respectively. Note that at –90 mV the current was reversibly blocked by 1 µM strychnine, indicating the direct involvement of α9α10‐nAChRs; at –40 mV, the outward current was prevented by the absence of Ca^2+^ in the extracellular solution, indicating the presence of SK2 channels. *B*, same experiments as in panel *A* but performed in apical‐coil OHCs from *Ca_V_1.3^−/−^* mice. Note that ACh produced very little or no responses at both potentials. *C* and *D*, maximum intensity projections of confocal *z*‐stack images that were taken from mature P11 apical (*C*) and basal coil (*D*) of wild‐type and *Ca_V_1.3^−/−^* mice. Immunostaining for SK2 channels (green) and ChAT, which is used to visualize the efferent olivocochlear innervation of OHCs (red); Myo7a (blue) was used as the hair‐cell marker. Scale bars: 10 µm. *E–H*, whole‐cell voltage‐clamp recordings obtained from mature OHCs in wild‐type (*E*) and *Ca_V_1.3^−/−^* (*F–H*) mice during the superfusion of 40 mm extracellular K^+^. Lower panels in *E* and *H* show an expanded time scale of the area shown in the panels above. *I*–*K*, maximum intensity projections of confocal *z*‐stack images taken at two different frequencies along the cochlea (apical: 8 kHz; basal: 32 kHz) from P11 wild‐type (*J*) and *Ca_V_1.3^−/−^* mice (*K*). Immunostaining for the OHC marker prestin (*I*: green) and ChAT (*I*: white), which is used to visualize the efferent terminals and fibres below the IHCs, tunnel crossing fibres (arrows), and terminals below the OHCs. In the cochlear apical coil of *Ca_V_1.3^−/−^* mice (*K*) there were fewer ChAT‐labelled tunnel crossing fibres and OHC terminals than wild‐type mice (*J*). The ChAT‐labelled OHC terminals at 8 kHz were also disordered and larger than in the wild type. In the basal coil, the efferent innervation was visually comparable between the two genotypes. Scale bars: 10 µm.

Wild‐type apical OHCs expressed nAChRs since they responded to the extracellular application of ACh with a large inward current at the holding potential of −90 mV, which was blocked by the specific α9α10‐nAChRs antagonist strychnine (–256 ± 53 pA, *n* = 4, at –90 mV: Fig. 7 *A*, top). The presence of SK2 channels was indicated by ACh‐induced outward current (200 ± 6 pA, *n* = 3: Fig. 7 *A*, bottom), when cells were held at −40 mV, that was removed by the perfusion of a Ca^2+^‐free solution, as previously described (Glowatzki & Fuchs, 2000; Oliver *et al*. 2000; Marcotti *et al*. 2004a). In apical OHCs from *Ca_V_1.3^−/−^* mice, ACh elicited very small responses both at –90 mV (–6.6 ± 4.2 pA, *n* = 5: Fig. 7 *B*, top) and –40 mV (7.2 ± 4.9 pA, *n* = 5: Fig. 7 *B*, bottom), indicating that the expression of both nAChRs and SK2 channels is either absent or reduced. Immunolabelling experiments in apical coil OHCs confirmed that SK2 channels were present in wild‐type but almost completely absent in *Ca_V_1.3^−/−^* mice (Fig. 7 *C*). Moreover, efferent cholinergic terminals on P11 OHCs, which were visualized by ChAT immunoreactivity, were very few and far between in *Ca_V_1.3^−/−^* compared to wild‐type mice (Fig. 7 *C*), indicating that cholinergic fibres themselves are also strongly affected by the absence of Ca_V_1.3 channels in apical OHCs. In the basal coil both the pre‐ and postsynaptic elements were present in most OHCs from *Ca_V_1.3^−/−^* mice, although SK2 puncta appeared less prominent than in wild‐type mice (Fig. 7 *D*).

In order to establish whether the few remaining efferent terminals on OHCs from *Ca_V_1.3^−/−^* mice were functional, we perfused the cochlea with a high‐K^+^ extracellular solution, which depolarizes the efferent synaptic terminals, thereby triggering the release of ACh onto OHCs. Under whole‐cell patch clamp, the superfusion of 40 mm KCl caused a sustained, inward current in OHCs from wild‐type mice (Fig. 7 *E*), owing to a positive shift in the K^+^ reversal potential in these cells. This current was followed by high‐frequency synaptic currents (Oliver *et al*. 2000; Lioudyno *et al*. 2004), which were caused by the activation of nAChRs following the release of ACh from the efferent terminals onto OHCs. These spontaneous currents were recorded in all 7 control OHCs tested; in cells with stable recordings lasting > 1 min, the average amplitude of the inward currents was 227 ± 3 pA (*n* = 4, P11). In *Ca_V_1.3^−/−^* mice, 8 out of 12 OHCs tested remained either silent (5 cells: Fig. 7 *F*) or showed a very minimal activity (3 cells: Fig. 7 *G*) during the application of 40 mm K^+^. The 4 remaining OHCs responded to high K^+^ with more spiking activity (Fig. 7 *H*), although the amplitude of the inward currents was significantly smaller than that measured in wild‐type OHCs (167 ± 6 pA, *n* = 4, P10–P11, *P < *0.0001, *t* test) than that measured in wild‐type OHCs. We further assessed the cholinergic innervation with ChAT‐immunoreactivity (Fig. 7 *I–K*). Compared to wild‐type mice (Fig. 7 *J*), the apical cochlear coil from *Cav1.3^−/−^* mice showed highly disorganized ChAT‐labelled efferent fibres, but progressively less so towards the base (32 kHz: Fig. 7 *K*). *Ca_V_1.3^−/ −^* mice also had very dense ChAT labelling below the IHCs, with tunnel crossing fibres showing an irregular pattern of large terminals on OHCs (8 kHz: Fig. 7 *K*).

The above experiments testing the efferent synapses and fibres indicate that the majority of apical coil OHCs from *Ca_V_1.3^−/−^* mice do not acquire the characteristic cholinergic efferent innervation. The innervation of basal OHCs appeared less affected by the absence of Ca_V_1.3 Ca^2+^ channels.

### Basal OHCs from *Ca_V_1.3^−/−^* mice exhibit a milder phenotype

**Figure 8:**
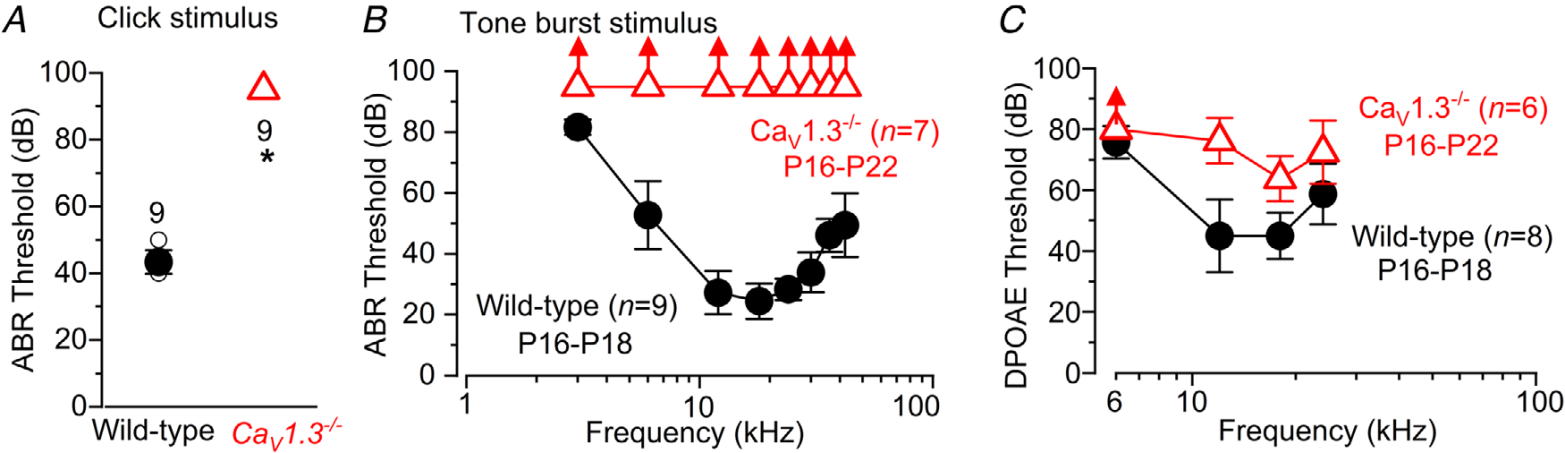
DPOAE, but not ABR, thresholds are still present in the high‐frequency region of *Ca_V_1.3^‐/^^−^* mice *A* and *B*, auditory brainstem responses (ABRs) for click (*A*) and tone burst stimuli (*B*) in P16–P18 wild‐type (circles) and P16–P22 *Ca_V_1.3^−/−^* mice (triangles) as a function of frequency position along the cochlea (3, 6, 12, 18, 24, 30, 36, 42 kHz). *Ca_V_1.3^−/−^* mice were profoundly deaf at all cochlear frequencies tested. *C*, DPOAE thresholds (6, 12, 18, 24 kHz) from P16–P18 wild‐type and P16–P22 *Ca_V_1.3^−/−^* mice. Data are means ± SD.

The above data suggest that the absence of the Ca_V_1.3 Ca^2+^ channels during early postnatal stages prevent the full maturation of apical OHCs. Although basal OHCs appeared to develop normally, these conclusions are only made from immunolabelling experiments, which do not provide a comprehensive physiological read‐out of OHC activity. Moreover, it has been shown that even basal OHCs will eventually degenerate in *Ca_V_1.3^−/−^* mice before ∼P30 (Platzer *et al*. 2000). Therefore, we performed *in vivo* experiments to primarily test the function of OHCs. Initially, we tested the hearing ability of the *Ca_V_1.3^−/−^* mice by recording auditory brainstem responses (ABRs), which provide a measure of the activity of the auditory neurons downstream of IHCs. We found that, compared to wild‐type mice, which had thresholds as low as 40 dB, *Ca_V_1.3^−/−^* mice showed threshold above 95 dB for both click and pure‐tone evoked ABRs (Fig. 8 *A* and *B*), which is consistent with an absence of Ca_V_1.3 Ca^2+^ channel‐induced neurotransmitter release from the IHCs (Brandt *et al*. 2003) and also with previous observations (Platzer *et al*. 2000; Eckrich *et al*. 2019). We then performed distortion product otoacoustic emissions (DPOAEs), which are read‐outs of amplification and cochlear non‐linearities, which are induced by the displacement of OHC stereociliary bundles during sound‐induced motion of the cochlear partition. We found that the DPOAE thresholds, for frequencies between 6 kHz and 30 kHz in P16–P22 *Ca_V_1.3^−/−^* mice were significantly elevated compared to P16–P18 wild‐type mice (*P* < 0.0001, two‐way ANOVA). We found that DPOAE thresholds above noise floor were still present in the high‐frequency regions, although significantly elevated compared to those in wild‐type mice (12 kHz and 18 kHz: *P < *0.0001; 24 kHz: *P = *0.0055; Tukey's *post hoc* test).This result indicates that the biophysical properties of basal coil‐OHCs are also affected by the absence of the Ca_V_1.3 Ca^2+^ channels, although to a lesser extent than those of apical cells.

### IHCs from Ca*_V_1.3^−/−^* mice have similar maturation defects to OHCs

**Figure 9:**
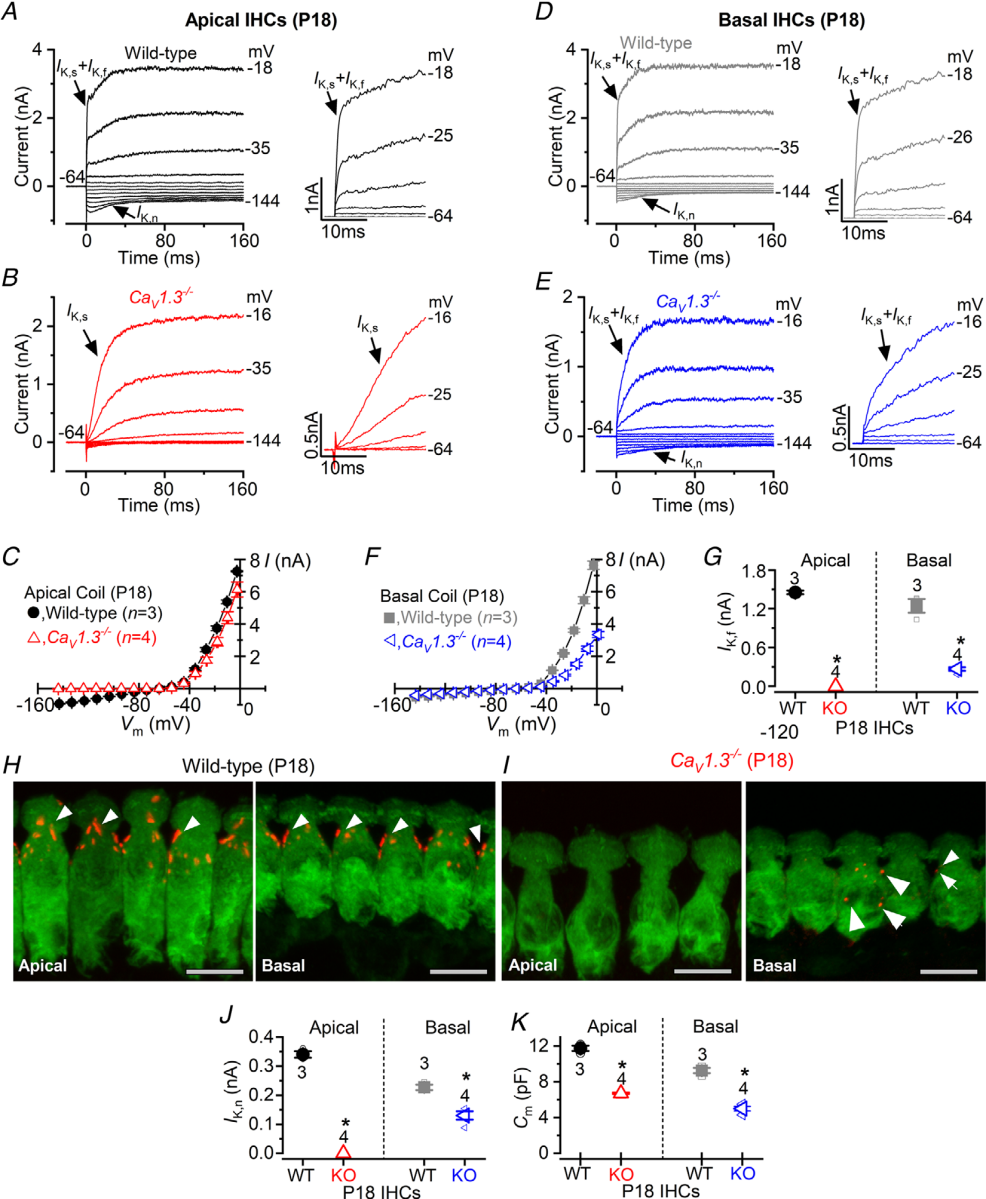
Potassium current expressed in post‐hearing IHCs of *Ca_V_1.3^−^^/^^−^* mutant mice *A* and *B*, potassium currents recorded from P18 IHCs of the apical‐coil of the cochlea of wild‐type (*A*) and *Ca_V_1.3^−/−^* mutant mice (*B*) using 10 mV depolarizing voltage steps from –64 mV to the various test potentials shown by some of the traces. The adult‐type currents (*I*_K,f_ and *I*_K,n_) were only present in IHCs from wild‐type mice (*A*). IHCs from *Ca_V_1.3^−/−^* mice retained the delayed rectifier current (*B*, *I*_K_) characteristic of immature cells (Marcotti *et al*. 2003a). The absence of the rapidly activating *I*_K,f_ in *Ca_V_1.3^−/−^* IHCs is also evident when comparing the activation time course of the total outward currents on an expanded time scale (see insets). *C*, average current‐voltage (*I‐V*) curves obtained from apical P18 IHCs of wild‐type and *Ca_V_1.3^−/−^* mice. *D* and *E*, potassium currents recorded from P18 IHCs of the basal‐coil of the cochlea of wild‐type (*D*) and *Ca_V_1.3^−/−^* mice (*E*) as described in panels *A* and *B*. Different from apical IHCs of *Ca_V_1.3^−/−^* mice (*B*), basal cells retain *I*_K,n_ and some *I*_K,f_ (*E*). *F*, average current‐voltage curves obtained from P18 basal IHCs of wild‐type and *Ca_V_1.3^−/−^* mice. *G*, average size of *I*_K,f_ recorded from apical and basal IHCs of both genotypes. The isolated *I*_K,f_ was measured as previously described (Marcotti *et al*. 2003a: current measured at 1.5 ms from the current onset and at the membrane potential of −25 mV). *H* and *I*, maximum intensity projections of confocal *z*‐stack images of IHCs taken from mature P18 wild‐type (*H*) and *Ca_V_1.3^−/−^* mice (*I*) of both apical (left panels) and basal (right panel) coil of the cochlea. Immunostaining for BK channels (arrowheads) and Myo7a (IHC marker), which is used to visualize the IHCs. Note that BK puncta in IHCs from *Ca_V_1.3^−/−^* mice were either absent in apical (*I*, left panel) or very few in basal cells (*I*, arrows in right panel). Scale bars: 10 µm. *J*, average size of *I*_K,n_ recorded from apical and basal IHCs of both genotypes. The isolated *I*_K,n_ was measured as previously described (Marcotti *et al*. 2003a: difference between the peak and steady‐state deactivating tail current at the membrane potential of −124 mV). *K*, average IHC membrane capacitance (*C*_m_) in both genotypes and as a function of cochlear position. In panels *G, J* and *K*, single cell value recordings (open symbols) are also plotted behind the average closed symbols. ^*^Statistical significance (see Results).

It is well established that Ca_V_1.3 Ca^2+^ channels contribute to ∼90% of the total Ca^2+^ current in apical coil IHCs and OHCs (Platzer *et al*. 2000; Brandt *et al*. 2003; Beurg *et al*. 2008). Since electrophysiological recording from mature basal‐coil IHCs can be reliably performed (e.g. Marcotti *et al*. 2004b), we investigated whether a differential regulation in development was also present between apical and basal IHCs of *Ca_V_1.3^−/−^* mice (Fig. 9 *A–K*). Mature IHCs express the characteristic rapid‐activating Ca^2+^‐activated K^+^ current *I*_K,f_ (Kros *et al*. 1998; Marcotti *et al*. 2004b) and, similar to OHCs, *I*_K,n_ (Oliver *et al*. 2003; Marcotti *et al*. 2003a) from about the onset of hearing at P12. We found that, in agreement with previous findings (Brandt *et al*. 2003; Eckrich *et al*. 2019), P18 apical IHCs from *Ca_V_1.3^−/−^* mice failed to acquire both *I*_K,f_ and *I*_K,n_ (Fig. 9 *A–C* and *G*). In P18 basal IHCs of *Ca_V_1.3^−/−^* mice, *I*_K,f_ was present but significantly reduced in size compared to that recorded in wild‐type cells (Fig. 9 *D–G*; *P < *0.0002). These electrophysiological data are supported by immunolabelling experiments (Fig. 9 *H* and *I*) showing that the expression of the BK channels (carrying *I*_K,f_) at the neck region of IHCs (Pyott *et al*. 2004), was either absent (apical IHCs) or strongly reduced (basal IHCs) in *Ca_V_1.3^−/−^* mice (Fig. 9 *I*) compared to wild‐type cells (Fig. 9 *H*). Similar to *I*_K,f_, *I*_K,n_ was absent in apical IHCs of *Ca_V_1.3^−/−^* mice, but present in basal cells albeit again significantly reduced compared to that recorded in IHCs of wild‐type mice (Fig. 9 *D–F* and *J*; *P = *0.0037). We also found that the cell membrane capacitance *C*_m_ is significantly reduced in both apical and basal IHCs of *Ca_V_1.3^−/−^* mice compared to that measured in cells from wild‐type mice (Fig. 9 *K: P > *0.001 for both apical and basal IHCs; Tukey's *post hoc* test); the reduced *C*_m_ was also evident from the immunolabelling images (Fig. 9 *H* and *I*). When the size of *I*_K,f_ and *I*_K,n_ was normalized to the *C*_m_ of IHCs, only the former was still significantly larger in basal IHCs of wild‐type compared to that of *Ca_V_1.3^−/−^* mice (*I*_K,f_: *P* = 0.0002; *I*_K,n_: *P* = 0.6639).

A smaller *C*_m_ was also seen in apical OHCs of *Ca_V_1.3^−/−^* mice at P13 (Fig. 2 *E*), but information about basal OHCs is missing due to our inability to perform reliable electrophysiological recordings from these cells (see above). In order to have an indication of the size of OHCs in the basal coil, we estimated the hair cell volume by measuring pixel area from *z*‐stack immunofluorescence images (see Methods). Initially we confirmed the reliability of the above imaging method by comparing the measured cell volume with known surface‐area (*C*_m_) measurements recorded using patch clamp electrophysiology. Although the two techniques measure different cellular characteristics, they both provide an indication of cell size. We found that the volume of basal IHC was reduced by ∼33% between P11 *Ca_V_1.3^−/−^* (301 ± 24 µm^3^, *n = *25 cells) and age‐matched wild‐type mice (450 ± 22 µm^3^, *n = *32, *P* < 0.0001), which is consistent with our *C*_m_ measurements (∼46%: Fig. 9 *K*). We found that the volume of basal OHCs at P11 in *Ca_V_1.3^−/−^* mice was also significantly reduced by ∼30% (109 ± 6 µm^3^, *n = *83 cells) compared to that of age‐matched wild‐type mice (156 ± 4 µm^3^, *n = *76, *P* < 0.0001), confirming that the reduced size of mature hair cells of *Ca_V_1.3^−/−^* mice is a general characteristic associated with the absence of Ca_V_1.3 Ca^2+^ channels.

These results indicate that the absence of Ca_V_1.3 Ca^2+^ channels during pre‐hearing stages of development differentially affected the developmental acquisition of the mature biophysical profile of apical and basal IHCs, with the latter being less influenced by early Ca^2+^ signals, as seen for OHCs (see above). One common change observed in hair cells of *Ca_V_1.3^−/−^* mice irrespective of their cochlear position was the reduced surface area.

### The differential maturation of apical and basal hair cells in *Ca_V_1.3^−/−^* mice is not due to tonotopic differences in the residual Ca^2+^ current

The above findings indicate that the maturation of both OHCs and IHCs from the basal cochlea of *Ca_V_1.3^−/−^* mice was affected by the absence of Ca_V_1.3 Ca^2+^ channels, but to a lesser extent compared to apical cells. Since ∼10% of the total Ca^2+^ current in apical hair cells has been shown to be carried by Ca^2+^ channels other than Ca_V_1.3 (Platzer *et al*. 2000; Michna *et al*. 2003), we sought to investigate whether basal cells express a larger residual Ca^2+^ current that could explain the milder phenotype of these cells in *Ca_V_1.3^−/−^* mice. Although the identity of the residual Ca^2+^ channels in auditory hair cells is still unknown (for a recent review see Pangrsic *et al*. 2018), it could involve Ca_V_1.4 (Brandt *et al*. 2003) and/or Ca_V_3.1 T‐type subunits (Inagaki *et al*. 2008; Levic & Dulon, 2012).

**Figure 10:**
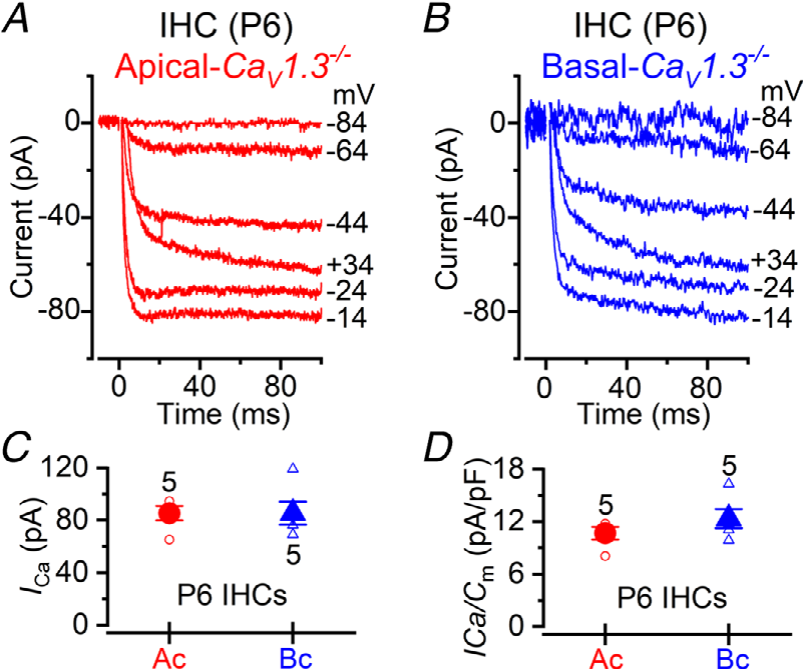
Residual Ca^2+^ current in IHCs of *Ca_V_1.3^−/−^* mice *A* and *B*, calcium currents recorded from apical (*A*) and basal (*B*) IHCs from P6 *Ca_V_1.3^−/−^* mice. Currents were elicited by depolarizing voltage steps of 10 mV increments (100 ms in duration) starting from the holding potential of –84 mV. For clarity only some of the traces are shown. Actual test potentials, corrected for voltage drop across uncompensated *R*_s_, are shown next to the traces. Residual capacitative transients have been blanked. *C*, comparison of the peak *I*_Ca_ in apical (circles) and basal (triangles) IHCs. *D*, same data as in panel *C*, but normalized to the IHC membrane capacitance (*C*_m_). Recordings were obtained near body temperature (34–37°C) and using 1.3 mm extracellular Ca^2+^.

We performed whole‐cells recordings to measure the Ca^2+^ currents in P6 IHCs from *Ca_V_1.3^−/−^* mice (Fig. 10 *A–D*), a time when spontaneous Ca^2+^‐dependent action potentials are present in IHCs of wild‐type mice (e.g. Marcotti *et al*. 2003b; Johnson *et al*. 2011b). Recordings were performed at body temperature and using physiological 1.3 mm extracellular Ca^2+^ (Johnson *et al*. 2005). We found that the residual Ca^2+^ current in IHCs of *Ca_V_1.3^−/−^* mice showed no signs of inactivation during the 100 ms voltage steps, indicating that it is unlikely to be carried by T‐type Ca^2+^ channels (Zamponi *et al*. 2015). The size of the residual Ca^2+^ current was indistinguishable between the low‐ (apical) and high‐frequency (basal) cells (*P* = 0.994, Fig. 10 *C*), even when it was normalized to the respective *C*_m_ (*P* = 0.239, Fig. 10 *D*). Although we did not investigate the size of the Ca^2+^ current in OHCs of *Ca_V_1.3^−/−^* mice with electrophysiology, which in the presence of physiological 1.3 mm extracellular Ca^2+^ is likely to be in the order of a few picoamps (Michna *et al*. 2003), 2‐photon imaging experiments (Fig. 1 *G* and *I*) show that both apical and basal OHCs of *Ca_V_1.3^−/−^* mice are completely deprived of any spontaneous Ca^2+^ signals. This indicates that, as for IHCs of *Ca_V_1.3^−/−^* mice (Fig. 10), the residual Ca^2+^ current in OHCs is likely to be similar in the two cochlear locations, and as such is unlikely to contribute to the differential regulation of hair cell development.

## Discussion

We have found that the intrinsic spiking activity driven by Ca_V_1.3 Ca^2+^ channels in early postnatal hair cells differentially regulates their maturation along the cochlea. OHCs and IHCs acquire their characteristic mature profile from ∼P7 and ∼P12, respectively, with the upregulation of several basolateral membrane ion channels and, for OHCs, the motor protein prestin. Like IHCs (see below), immature OHCs transiently generate spontaneous Ca^2+^‐dependent APs under near‐physiological recording conditions. We found that these APs regulate the normal temporal acquisition of mature‐like basolateral membrane currents in low‐frequency apical hair cells. This includes *I*_K,n_, which is carried by KCNQ4 channels and the current associated with the inhibitory cholinergic efferent system (*I*_ACh_, carried by α9α10‐nAChRs, and *I*_SK2_) in OHCs, and *I*_K,n_ and *I*_K,f_ (BK channels) in IHCs (see also Brandt *et al*. 2003). OHC electromotility was not affected by the ablation of APs *in vivo* using *Ca_V_1.3^−/−^* mice. Different from apical cells, the maturation of high‐frequency basal hair cells from *Ca_V_1.3^−/−^* mice was found to be less dependent on Ca^2+^ APs, since mature‐like proteins were present in their basolateral membrane, but to a lesser degree compared to wild‐type mice. The hair cell size in *Ca_V_1.3^−/−^* mice was significantly smaller than that of wild type, irrespectively of cochlear position. We propose that the development of low‐ and high‐frequency hair cells of the mammalian cochlea is differentially regulated during pre‐hearing stages, with apical hair cells being more strongly dependent on experience‐independent Ca^2+^ AP activity.

### Action potentials in cochlear hair cells

In the mammalian cochlea, Ca^2+^‐dependent APs have been shown to occur spontaneously in immature IHCs (Kros *et al*. 1998; Johnson *et al*. 2011b, 2017; Sendin *et al*. 2014; Corns *et al*. 2018; Eckrich *et al*. 2018) when experiments are performed under near‐physiological recording conditions. Here we have shown that reliable and long‐duration recordings of AP activity in early postnatal OHCs require experimental conditions that preserve the *in vivo* intracellular milieu and Ca^2+^ levels (cell‐attached patch clamp). Under these conditions, APs in OHCs exhibit a higher frequency in the basal (∼2 Hz) than in the apical (∼0.8 Hz) turn of the cochlea, similar to previous observations from immature IHCs (Johnson *et al*. 2011b, 2017). These APs are driven by Ca_V_1.3 Ca^2+^ channels, which represent > 90% of the Ca^2+^ channels expressed in hair cells (Platzer *et al*. 2000; Michna *et al*. 2003), but there are notable differences between the activity of IHCs and OHCs. While OHCs showed a bursting pattern irrespective of their cochlear location, IHCs exhibit an apex‐to‐base difference, with apical cells showing a bursting firing pattern and basal IHCs firing a more regular activity (Johnson *et al*. 2011b). These different AP characteristics between the two sensory cells could be due to the fact that only immature IHCs, but not OHCs, contribute directly to the firing activity of type I spiral ganglion neurons (Jones *et al*. 2007; Tritsch *et al*. 2010) and to the tonotopic refinement of sensory maps along the auditory pathway in the developing brainstem (Lippe, 1995; Clause *et al*. 2014). Furthermore, while AP activity in OHCs only occurs during the first few days after birth, that of IHCs persists throughout pre‐hearing stages of postnatal development (Johnson *et al*. 2012), reflecting the different roles of the two cell types in auditory pathway development.

### Role of action potentials in the maturation of hair cells

Functionally mature OHCs possess both sensory and motor functions, which allows them to sharpen the frequency selectivity of IHCs along the cochlear partition (Ashmore, 2008). This requires specific biophysical and morphological properties that are rapidly acquired at the onset of maturation towards the end of the first postnatal week (Corns *et al*. 2014), 5 days before the IHCs mature at the onset of hearing at ∼P12 (Ehret, 1975). Crucial to their function is the acquisition of the negatively activating delayed rectifier K^+^ current *I*_K,n_, which flows through KCNQ4 channels (Kubisch *et al*. 1999), and in mice first appears at about P8 in OHCs (Marcotti & Kros, 1999) and at P12 in IHCs (Oliver *et al*. 2003; Marcotti *et al*. 2003a). OHC maturation is also associated with the onset of somatic motility at P7–P8 (Marcotti & Kros, 1999; Abe *et al*. 2007), which is driven by the motor protein prestin (Zheng *et al*. 2000; Liberman *et al*. 2002), and the formation of connections with cholinergic efferent and type II afferent fibres (Echteler, 1992; Simmons, 1994; Simmons *et al*. 1996). The medial olivocochlear (MOC) cholinergic efferent system represents the primary innervation of mature OHCs, and its correct function requires the expression of small conductance Ca^2+^‐activated K^+^ channels (SK2) and α9α10‐nAChRs on the postsynaptic membrane (Oliver *et al*. 2000; Lioudyno *et al*. 2004). Unlike the type I fibres contacting IHCs, type II afferent fibres seem to be activated only by sounds loud enough to cause acoustic trauma (Flores *et al*. 2015; Liu *et al*. 2015).

The development of sensory systems is normally influenced by transient periods of experience‐independent Ca^2+^ action potential activity occurring just before the onset of function (Katz & Shatz, 1996; Huberman *et al*. 2008; Blankenship & Feller 2010). This Ca^2+^‐dependent activity has also been shown to regulate changes in channel expression (Moody & Bosma, 2005). Here we show that immature OHCs transiently fire spontaneous Ca^2+^ APs during the first few days after birth, which can also be visualized as rapid Ca^2+^ signals under 2‐photon imaging (Fig. 1; see also Ceriani *et al*. 2019). In *Ca_V_1.3^−/−^* mice, in which Ca^2+^ APs are abolished, we found impaired expression of the mature basolateral membrane ion channels that are crucial for hair cell function. In IHCs, the KCNQ4 and BK channels were absent in apical cells but expressed at a low level in basal IHCs. In apical OHCs, the KCNQ4 channels and those required postsynaptically for the efferent function (SK2 and α9α10‐nAChRs) were also largely reduced or absent. Although we could not provide a similar electrophysiological quantification for basal OHCs, DPOAE recordings (Fig. 8) indicated that the maturation of these cells is likely to be affected in *Ca_V_1.3^−/−^* mice, although like basal IHCs, to a lesser extent than apical cells. It is well established that both the size of IHCs and OHCs in mice increases during development (Marcotti & Kros, 1999; Marcotti *et al*. 2003a), a process that is largely prevented in *Ca_V_1.3^−/−^* mice irrespective of hair cell position along the cochlea. Since the size and kinetics of the residual Ca^2+^ current in IHCs of *Ca_V_1.3^−/−^* mice are comparable between apical and basal cells, it is possible that the milder phenotype in high‐frequency IHCs is due to their development being under a ‘stronger’ activity‐independent genetic influence.

The substantially reduced number of efferent contacts on pre‐hearing OHCs of *Ca_V_1.3^−/−^* mice, together with the reduced expression of the postsynaptic SK2 channels and α9α10‐nAChRs, could potentially be due to the absence of Ca_V_1.3 channels in the auditory brainstem (Hirtz *et al*. 2011). However, this seems unlikely to be the case since the MOC neurons have been shown to make normal synaptic contacts with developing IHCs in P15 *Ca_v_1.3^−/−^* mice (Glueckert *et al*. 2003). This indicates that the inability of OHC efferent synapses to mature is likely to be a direct consequence of an absence of AP activity during early stages of postnatal development. A reduction in the number of OHC synaptic ribbons has been linked to a decreased synchronization of AP activity between adjacent OHCs, which occurs when the electrical connectivity between non‐sensory cells in the cochlea is disrupted (Ceriani *et al*. 2019). The adult‐like characteristic of OHCs that was not influenced by spontaneous Ca^2+^ APs was their electromotility, suggesting that the expression of the motor protein prestin is most probably controlled by an intrinsic genetic programme. This is also supported by recent evidence that the transcription factor *Ikzf2* is required for the normal expression of prestin (Chessum *et al*. 2018).

Although spontaneous Ca^2+^ AP activity has been shown to be crucial for hair‐cell maturation, genetic programmes seem to play a crucial role early on during their functional development. The combination of these two independent but highly regulated developmental mechanisms ensures that the highly specialized features of IHCs and OHCs are optimal to process sound information along the tonotopic axis of the mammalian cochlea.

### OHCs of *Ca_V_1.3^−/−^* mice degenerate with the onset of hearing

Our morphological (OHC size and bundle appearance) and physiological data (resting *V*_m_, electromotility and expression of the main Ca^2+^ binding protein oncomodulin) demonstrate that pre‐hearing OHCs from *Ca_V_1.3^−/−^* mice, despite being ‘stuck’ at an intermediate developmental profile, are physiologically viable and healthy, and they only begin to rapidly degenerate upon the onset of hearing. A similar level of OHC degeneration, after the onset of hearing, has previously being reported for mutations in *Tmc1* (Marcotti *et al*. 2006), one of the main candidates for the mechanoelectrical transducer channel (Kawashima *et al*. 2011; Corns *et al*. 2016; Ballesteros *et al*. 2018; Pan *et al*. 2018). Similar to our findings in *Ca_V_1.3^−/−^* mice, an absent or mutated *Tmc1* in OHCs does not affect their electromotility but does prevent them acquiring *I*_K,n_ at P8, causing them to retain an immature basolateral current profile at hearing onset (Marcotti *et al*. 2006). *I*_K,n_ plays a key role in mature OHCs since it provides the majority of the outward K^+^ current and is nearly fully activated (∼80%: Marcotti & Kros, 1999) at their resting *V*_m_ (near −40 mV: Johnson *et al*. 2011a). As such, *I*_K,n_ provides the major exit route for K^+^ ions that have entered OHCs via the opening of the mechanoelectrical transducer (MET) channels during sound stimulation. The reduced *I*_K,n_ in OHCs of *Ca_V_1.3^−/−^* mice would lead to intracellular K^+^ accumulation as soon as their mechanoelectrical transducer apparatus is stimulated by incoming sound, leading to their abrupt degeneration. In contrast to OHCs, IHCs of *Ca_V_1.3^−/−^* mice do not degenerate until ∼P30 (Platzer *et al*. 2000), most likely because of their smaller resting MET current (Johnson *et al*. 2011a) and the fact that they still express a large outward K^+^ current.

We propose that the mammalian cochlea uses distinct mechanisms to orchestrate the maturation of the sensory epithelium during pre‐hearing stages of development. Intrinsic Ca^2+^‐dependent AP activity in OHCs is restricted to the first postnatal week and is required for the normal expression of the basolateral ion channels characteristic of mature apical, low‐frequency OHCs from ∼P7 onwards (*I*_K,n_, SK2 and α9α10‐nAChRs). Moreover, waves of Ca^2+^ activity in the non‐sensory greater epithelial ridge synchronize the AP firing of apical OHC, resulting in the refinement of their afferent innervation (Ceriani *et al*. 2019). We also found that low‐frequency, apical‐coil OHCs are more strongly dependent on experience‐independent Ca^2+^ AP activity than high‐frequency cells. Moreover, APs are not required for the functional acquisition of the highly specialized electromotile activity in OHCs, which is regulated by the transcription factor *Ikzf2* (Chessum *et al*. 2018). The effect of Ca^2+^ activity on IHCs, which occurs throughout post‐natal prehearing stages (Johnson *et al*. 2011b, 2012), has functionally different consequences for the cochlea since it seems only to contribute to the maturation of the basolateral membrane properties of the cells themselves (Brandt *et al*. 2003; Johnson *et al*. 2013; Corns *et al*. 2018). However, similar to OHCs, the development of high‐frequency IHCs is also less affected by the absence of experience‐independent Ca^2+^ signals. The increase in hair cell size during development appears to be the only feature that is similarly regulated by early Ca^2+^ activity, irrespective of cell position along the cochlea. Finally, only the Ca_V_1.3 Ca^2+^ channels, but not the channels responsible for the residual Ca^2+^ current in *Ca_V_1.3^−/−^* mice (possibly Ca_V_1.4: see Results section), exert a key role in the maturation of hair cells. Our results reveal that an extraordinary subtlety between genetic and physiological regulation is orchestrated to ensure the correct tonotopic differentiation of hair cells along the cochlea, allowing coherent functional development of the adult auditory system.

## Additional information

### Competing interests

The authors declare no conflict of interest.

### Author contributions

The majority of the experiments were conducted at the University of Sheffield. Experiments for Fig. 4 were conducted at the University of Sussex and those for Fig. 7 *I–K* at Baylor University. J‐Y.J., F.C. A.H., S.L.J., P.Y., D.D.S., C.J.K. and W.M. were involved in the acquisition, analysis or interpretation of data for the work. J‐Y.J, F.C., C.J.K. and W.M. were involved in drafting the work or revising it critically for important intellectual content. W.M. conceived and designed the study. All authors approved the final version of the manuscript. All authors agree to be accountable for all aspects of the work in ensuring that questions related to the accuracy or integrity of any part of the work are appropriately investigated and resolved. All persons designated as authors qualify for authorship, and all those who qualify for authorship are listed.

### Funding

This work was supported by the Wellcome Trust to W.M. (102 892/Z/13/Z). C.J.K. was supported by the MRC (MR/K005561/1). S.L.J. is a Royal Society URF.
